# Napabucasin overcomes cisplatin resistance in ovarian germ cell tumor-derived cell line by inhibiting cancer stemness

**DOI:** 10.1186/s12935-020-01458-7

**Published:** 2020-08-03

**Authors:** Silvia Schmidtova, Lambert C. J. Dorssers, Katarina Kalavska, Ad J. M. Gillis, J. Wolter Oosterhuis, Hans Stoop, Svetlana Miklikova, Zuzana Kozovska, Monika Burikova, Katarina Gercakova, Erika Durinikova, Michal Chovanec, Michal Mego, Lucia Kucerova, Leendert H. J. Looijenga

**Affiliations:** 1 Department of Molecular Oncology, Cancer Research Institute, Biomedical Research Center, University Science Park for Biomedicine, Slovak Academy of Sciences, Dubravska cesta 9, 845 05 Bratislava, Slovakia; 2 Translational Research Unit, Faculty of Medicine, Comenius University, Klenova 1, 833 10 Bratislava, Slovakia; 3 Department of Pathology, Laboratory for Experimental Patho-Oncology, Erasmus MC Cancer Institute, University Medical Center Rotterdam, Wytemaweg 80, 3015 CN Rotterdam, The Netherlands; 4 2nd Department of Oncology, Faculty of Medicine, Comenius University and National Cancer Institute, Klenova 1, 833 10 Bratislava, Slovakia; 5 Princess Maxima Center for Pediatric Oncology, Heidelberglaan 25, 3584 CS Utrecht, The Netherlands

**Keywords:** Yolk sac tumor, Cisplatin, Aldehyde dehydrogenase, Cancer stem cells

## Abstract

**Background:**

Cisplatin resistance of ovarian yolk sac tumors (oYST) is a clinical challenge due to dismal patient prognosis, even though the disease is extremely rare. We investigated potential association between cisplatin resistance and cancer stem cell (CSC) markers in chemoresistant oYST cells and targeting strategies to overcome resistance in oYST.

**Methods:**

Chemoresistant cells were derived from chemosensitive human oYST cells by cultivation in cisplatin in vitro. Derivative cells were characterized by chemoresistance, functional assays, flow cytometry, gene expression and protein arrays focused on CSC markers. RNAseq, methylation and microRNA profiling were performed. Quail chorioallantoic membranes (CAM) with implanted oYST cells were used to analyze the micro-tumor extent and interconnection with the CAM. Tumorigenicity in vivo was determined on immunodeficient mouse model. Chemoresistant cells were treated by inhibitors intefering with the CSC properties to examine the chemosensitization to cisplatin.

**Results:**

Long-term cisplatin exposure resulted in seven-fold higher IC_50_ value in resistant cells, cross-resistance to oxaliplatin and carboplatin, and increased migratory capacity, invasiveness and tumorigenicity, associated with hypomethylation of differentially methylated genes/promotors. Resistant cells exhibited increased expression of prominin-1 (CD133), ATP binding cassette subfamily G member 2 (ABCG2), aldehyde dehydrogenase 3 isoform A1 (ALDH3A1), correlating with reduced gene and promoter methylation, as well as increased expression of ALDH1A3 and higher overall ALDH enzymatic activity, rendering them cross-resistant to DEAB, disulfiram and napabucasin. Salinomycin and tunicamycin were significantly more toxic to resistant cells. Pretreatment with napabucasin resensitized the cells to cisplatin and reduced their tumorigenicity in vivo.

**Conclusions:**

The novel chemoresistant cells represent unique model of refractory oYST. CSC markers are associated with cisplatin resistance being possible targets in chemorefractory oYST.

## Background

Malignant ovarian germ cell tumors (MOGCTs) represent 2-3% of all ovarian tumors and generally appear in children and women of reproductive age [1–3]. Yolk sac tumor (YST), also known as endodermal sinus tumor, is the second most prevalent histologic subtype of MOGCTs [4], showing a poorer prognosis [5]. Serum alpha-fetoprotein (AFP) is a useful marker for YST, informative for monitoring response to chemotherapy and tumor recurrence [6, 7]. Treatment modalities for MOGCTs are based on those for testicular germ cell tumors (TGCTs) [8, 9], including surgery, possibly followed by platinum-based chemotherapy [10–13].

Despite the fact that most patients can be cured, some relapse. If occurring within 4–6 weeks of therapy, the cancers are considered platinum-resistant, with an extremely poor prognosis, of which data regarding further treatment is lacking [3].

Chromosomal aberrations have been considered informative to identify determinants of chemoresistance and survival [14]. The MOGCTs are mainly nondiploid (tetraploid, polyploid, or aneuploid), with gain of parts or the short arm of chromosome 12 similarly to TGCTs [15]. Using high thoughput data-analyses Dorssers et al*.* [16] confirmed that nonseminomatous TGCTs are initiated by whole-genome duplication, followed by chromosome copy number changes, and accumulation of low numbers of somatic mutations, even in therapy-resistant cases. In addition, DNA methylation changes can occur during acquisition of drug resistance [17, 18].

It has become evident that a subpopulation of tumor cells, referred to as “cancer stem cells (CSCs)”, determine tumor recurrence, metastasis, aggressiveness and therapy resistance [19]. CSCs can be identified by defined markers [20], and by using functional approaches based on biochemical activities, including high enzymatic activity of aldehyde dehydrogenase (ALDH) detoxifying enzyme or Hoechst 33342 efflux ability [21].

Treatment strategies targeting CSCs combined with conventional therapies might improve cancer cure compared to monotherapies [22, 23]. The present study extensively examines a newly derived cisplatin-resistant oYST cell line (NOY-1 CisR), including sensitivity to various platinum derivates, migratory abilities, gene expression (i.p. CSC markers), tumorigenicity in vivo, as well as RNAseq, microRNA and methylation (EPIC) profiling. Our data show that chemoresistance of NOY-1 CisR cells is associated with increased expression of CSC markers (CD133, ABCG2 and ALDH), reversible using salinomycin, tunicamycin or napabucasin.

## Methods

### Cells

Human YST cell line NOY-1 (catalog number: ENG101, FA: Kerafast; Nagoya Ovarian Yolk sec tumor cell line 1, originated from a 28 year old female) was purchased and used for the study within 3 years within purchase and it is the only commercially available cell line model of oYST. The cisplatin-resistant subclone (NOY-1 CisR) was derived by propagating the cells in increasing concentrations of cisplatin (Hospira UK Ltd, Warwickshire, UK) for 6 months as described in the Additional file 1. Cells were maintained in RPMI (GIBCO® Invitrogen, Carlsbad, CA) containing 10% FBS (GIBCO® Invitrogen, Carlsbad, CA), 10,000 IU/ml penicillin (Biotica, Part. Lupca, Slovakia), 5 μg/ml streptomycin, 2.5 μg/ml amphotericin, 2 mM glutamine (PAA Laboratories GmbH) and 10 μg/ml insulin. Cells were cultivated at 37 °C in humidified atmosphere and 5% CO_2_.

Human ovarian cancer cell lines SKOV-3 and A2780 (kindly provided by Dr. Toro, Cancer Research Institute BMC SAS, Bratislava) were cultured in high glucose (4.5 g/l) Dulbecco’s modified Eagle medium (DMEM; PAN Biotech, Germany) supplemented with 5% FBS, 10,000 IU/mL penicillin, 5 μg/ml streptomycin, 2.5 μg/mL amphotericin and 2 mM glutamine.

Human colon cancer cell line HT-29/EGFP and its chemoresistant derivative HT-29/EGFP/FUR (kindly provided by Dr. Durinikova, Cancer Research Institute BMC SAS, Bratislava) were maintained in high glucose DMEM supplemented with 10% fetal calf serum (FCS; Biochrom AG, Germany), 2 mM glutamine (PAA Laboratories GmbH, Austria) or GlutaMAX (Gibco by Life Technologies, USA), 10 μg/ml gentamicin (Sandoz, Germany) and 2.5 μg/ml amphotericin B (Sigma-Aldrich, USA). Human mesenchymal stromal cells (MSC, kindly provided by Dr. Miklikova, Cancer Research Institute BMC SAS, Bratislava) used in this study were propagated in low glucose (1.0 g/l) DMEM supplemented as described above [24–27].

3D multicellular spheroids were prepared in quadruplicates of 5 × 10^3^ NOY-1 or NOY-1 CisR cells and seeded into 96-well ultra-low attachment plates (Corning 7007, Corning Inc., NY, USA) in 100 μl of RPMI culture medium (as described in Additional file 1). Three days later, pictures of the spheroids were taken.

### Viability assays

Chemicals were purchased from Sigma-Aldrich if not stated otherwise.

Quadruplicates of cells were plated at 3 × 10^3^ cells/100 μl media per well and were seeded in 96-well white-walled plates (Corning Costar Life Sciences, Amsterdam, NL) overnight. Following drugs were used: cisplatin, oxaliplatin (Fresenius Kabi Oncology Plc., Hampshire, UK), carboplatin (Fresenius Kabi Oncology Plc.), salinomycin, tunicamycin, DEAB, disulfiram and napabucasin (Abcam, Cambridge, UK). For the evaluation of chemosensitivity, cells were seeded in 96-well plates overnight and treated with cisplatin (0.01–3 μg/ml), oxaliplatin (0.156–20 μg/ml), carboplatin (0.625–10 ng/ml), salinomycin (10–110 ng/ml), tunicamycin (100–200 ng/ml), DEAB (40–100 μg/ml), disulfiram (25–40 ng/ml) and napabucasin (0–0.217 μg/ml). Relative viability of the cells was determined by the CellTiter-Glo™ Luminescent Cell Viability Assay (Promega Corporation, Madison, WI) and evaluated by the LumiStar GALAXY reader (BMG Labtechnologies, Offenburg, Germany) after 6–7 days of treatment. Experiments were performed at least three times of which the representative result is shown. Values were expressed as means ± SD and IC_50_ values were calculated by CalcuSyn 1.1 software.

### Methylation profiling

Generation of methylation profiles of cell line genomic DNA isolated using ethanol precipitation was performed at the Erasmus MC Department of Pathology molecular diagnostics lab according to the Illumina protocols (EPIC). Copy number alterations were resolved using the Conumee package (Hovestadt V, Zapatka M. conumee: Enhanced copy-number variation analysis using Illumina DNA methylation arrays. R package version 1.6.0. https://www.bioconductororg/packages/conumee/. 2015). Differential methylation was identified using the RnBeads package (https://rnbeads.org) using “SWAN” for normalization [28].

### miRNA profiling

Total RNA was prepared using Trizol (Thermo Fisher, USA). miRNAs were converted into cDNA using the specific megaplex primers (ThermoFisher, PN: 4399966) and the reverse transcription kit (ThermoFisher, PN: 3466596) and quantitated on TaqMan Low Density Arrays (384-well Microfluids TLDA card A, ThermoFisher, PN: 4398965) on a TaqMan 7900HT Fast Real-Time PCR Machine using the supplier protocols (ThermoFisher, PN: 4399721). TaqMan miRNA array output data (sds-files) were uploaded in the ThermoFisher Cloud App (https://www.thermofisher.com/nl/en/home/digital-science/thermo-fisher-connect/all-analysis-modules.html) and analyzed using defined threshold settings for each individual miRNA. Cq values were exported, and globally normalized in Excel.

### Gene expression

Cultured cells were collected by trypsinization and total RNA was isolated by NucleoSpin® RNA II (Macherey–Nagel, Germany) and treated with RNase-free DNase (Qiagen, Hilden, Germany). Total RNA was subjected to control PCR to confirm the absence of genomic DNA contamination. RNA concentration and quality were determined by gel electrophoresis and spectrophotometrically at 260/280 nm using the NanoDrop ND-1000 Spectrophotometer (Thermo Scientific, USA).

RNA was reverse transcribed with RevertAid™ H minus First Strand cDNA Synthesis Kit (Thermo Fisher Scientific Inc., Massachusetts, USA). For quantitative PCR we used following protocol: activation step at 95 °C for 2 min, 40 cycles of denaturation at 95 °C for 15, 30 s annealing and polymerization at 60 °C and plate read for 5 s at 71 °C, followed by melt cycle. The PCR reaction mixture (15 μl) contained 1 μl cDNA (100 ng), 0.4 μl respective specific primers (10 pmol/μl), 6.1 μl water and 7.5 μl GoTaq® qPCR Master Mix (Promega Corporation, Madison, WI). qPCR reaction was performed on CFX96™ Real-Time PCR Detection System (BIO-RAD Laboratories, USA) and analyzed by Bio-Rad CFX Manager software version 1.6. *HPRT1* or *ACTB* gene expressions were taken as endogenous reference. Relative gene expression was calculated using the 2^–ΔΔCt^ method. The results were reported as the n‐fold change in gene of interest expression in the resistant cell line normalized to the endogenous control (*HPRT1* or *ACTB*) and relative to the control group (= 1). Data represent mean ± SEM of three independent experiments. The significance of fold changes in gene expression between groups was analyzed using Student's *t*‐test applied to the ΔCt values.

The primer sequences used for expression analysis are listed in Additional file 2. Table S1.

### RNAseq analysis

Total RNA was prepared using Trizol, DNase-treated (RNeasy Micro Kit, Qiagen, Germany), and quality verified using fragment analysis. The NEBNext Ultra Directional RNA Library Prep Kit for Illumina was used to process the samples. The sample preparation was performed according to the protocol "NEBNext Ultra Directional RNA Library Prep Kit for Illumina" (NEB #E7420). Briefly, rRNA was depleted from total RNA using the rRNA depletion kit (NEB# E6310). After fragmentation of the rRNA reduced RNA, a cDNA synthesis was performed. This was used for ligation with the sequencing adapters and PCR amplification of the resulting product. Clustering and DNA sequencing using the Illumina cBot and HiSeq 4000 was performed according to manufacturer's protocols. The experiments were performed at GenomeScan B.V., Plesmanlaan 1d, 2333 BZ, Leiden. Processing of RNA-seq data was performed using UCSC human genome build hg38 and GENCODE annotation release 28 (GRCh38). FASTQC (v0.11.5) [29] was applied on the paired-end FASTQ files for quality control, both before and after running trimmomatic (v0.36) [30], which removed TrueSeq adapter sequences. STAR (v2.5.3a) [31] was used as aligner, with 2-pass mapping for each sample separately. Mapping quality plot was generated and checked based on sambamba Flagstat (v0.6.7) statistics [32]. Count files, with the number of reads for each gene were created with subread FeatureCounts (v1.5.2) [33].

### Data integration

Output of differentially methylated genes and promoters of RnBeads and RNA read counts were merged in RStudio (Version 1.1.463; using R version 3.5.1) using the Ensemble Gene ID’s. UCSC LiftOver tool was applied to convert hg19 genome coordinates of the conumee package to hg38 coordinates (https://genome-euro.ucsc.edu/cgi-bin/hgLiftOver). Plots were created in RStudio using general plotting packages.

### α-F-actin immunostaining

Ten thousand of cells growing on microscopic slides for 72 h were fixed with 4% paraformaldehyde in PBS for 15 min at room temperature and permeabilized with 0.05% Triton-X100 in PBS for 15 min. After overnight incubation with anti-F-actin rhodamine conjugated antibody (1:500; Invitrogen, Life Technology, Slovakia), nuclei were counterstained with DAPI (1:500). Staining patterns were analyzed with a Zeiss fluorescent microscope (AxioImager. Z2, Metafer, Alogo, Ltd., Czech Republic) using Isis upgrade software for Metafer (Alogo, Ltd., Czech Republic).

### Migration assay

Thirty thousand of NOY-1 and NOY-1 CisR cells per well were plated in quadruplicates in ImageLock 96-well plates (Essen BioScience, UK) and let to adhere overnight. Confluent monolayers were wounded with wound making tool (Essen BioScience, UK), washed twice and supplemented with culture medium. Images were taken every two hours for next 48 h in the IncuCyte ZOOM™ Kinetic Imaging System (Essen BioScience, UK). Cell migration was evaluated by IncuCyte ZOOM™ 2013A software (Essen BioScience, UK) based on the relative wound density measurements and expressed as means of three independent experiments run in quadruplicates ± SD.

### Flow cytometry

#### Aldefluor assay

The ALDEFLUOR™ Kit (StemCell Technologies, Vancouver BC) was used to detect intracellular enzyme activity of ALDH. Samples were prepared according to manufacturer’s instructions and ALDH activity was analyzed using BD Canto II Cytometer (Becton Dicinson, USA). Dead cells were excluded from the analysis based on the DAPI (4′, 6-diamidino-2-phenylindole) staining. Data were analyzed by FCS Express program.

#### CD133 staining

Cells were cultivated in standard culture medium for 3 days. CD133-PE antibody (Miltenyi Biotec GmbH, Germany) was used at a 1:50 dilution and incubated for 15 min with 500.000 tumor cells per sample. Dead cells were excluded based on DAPI staining. Cells were analyzed using BD Canto II cytometer and FCS Express software was used for the evaluation.

### Clonogenic assay

NOY-1 CisR cells were seeded (500 cells per well in 96-well culture plates) and let to adhere in standard culture medium (control) or in medium containing 0.06 μg/ml napabucasin. After 24 h, cisplatin was added to the cells, and medium-only in control wells. Plates were incubated for 6 days, after which cells were washed and fresh medium was added for another 4 days. Then the cells were stained with May-Grünwald solution.

### Quail Chorioallantoic Membrane (CAM) Model

Fertilized Japanese quail (Coturnix japonica) eggs from a breeding colony (Laying Line 01, Institute of Animal Biochemistry and Genetics, Centre of Biosciences SAS) were incubated in a forced draught incubator at 37 °C and 60% relative humidity. *Ex ovo* culture was prepared on the embryonic development day 3 (ED3), when the surface of eggs was wiped with 70% ethanol in a sterile laminar flow hood. The eggs were opened, embryos were transferred into 6-well culture plates (TPP, Switzerland) and returned to humidified incubator for the next 4 days [34]. NOY-1 and NOY-1 CisR cells were trypsinized, counted (1 × 10^6^ cells per sample), a silicone ring (6 mm) was positioned on the CAM surface and the cell suspension was placed therein on ED7. After 72 h, CAMs with tumor cells were separated and fixed with 4% formaldehyde. Subsequently, 5 µm paraffin sections were prepared for H&E staining. Canon DS126291 camera was used for taking pictures of the CAMs.

### Animal studies

Six- to 8-week-old athymic nude mice (Balb/c-nu/nu, Charles River, Germany) or SCID beige mice (CD17 Cg‑Prkdscid Lystbg/Crl) were used in accordance with institutional guidelines under approved protocols. Project was approved by the Institutional Ethic Committee and by the national competence authority (State Veterinary and Food Administration of the Slovak Republic), registration No. Ro 1976/17-221 in compliance with Directive 2010/63/EU of the European Parliament and the European Council and Regulation 377/2012 for the protection of animals used for scientific purposes. It was performed in the approved animal facility (license No. SK UCH 02017).

For the tumorigenicity test, suspension of 2 × 10^5^ NOY-1 and NOY-1 CisR cells in 60 μl of extracellular matrix (ECM) mixture 1:1 (30 μl serum free RPMI medium, 30 μl ECM) was injected s.c. into the flanks, in total four tumors per group.

In an independent study of napabucasin in vivo, SCID mice were used. NOY-1 CisR cells were treated with 1 μg/ml cisplatin for six days (group A) or pretreated with 0.06 µg/ml napabucasin and then exposed to 1 μg/ml cisplatin for six days (group B). Suspension of 2 × 10^5^ cells (from each group) in 100 μl of ECM mixture 1:1 (50 μl serum free RPMI medium, 50 μl ECM) was injected s.c. into the flanks, in total six tumors per group A and four tumors per group B.

In both experiments, tumors were measured by caliper and volume was calculated according to the formula for the volume of ellipsoid: ${\text{volume }} = \, 0.{\text{52 x }}\left( {\left( {{\text{width}} + {\text{lenght}}} \right)/{2}} \right)^{{3}}$. Animals were sacrificed at the point when the tumors exceeded 1 cm in diameter. The results were evaluated as the mean of tumor volume or tumor weight.

### Immunohistochemical staining

Serial 4 μm sections of formalin fixed paraffin embedded (FFPE) tissue were mounted on adhesive glass slides, and deparaffinized according BenchMark Ultra protocol. Antigen retrieval was performed with CC1 antigen retrieval solution (ref. 950-124) and Protease3 (ref. 760-2020). Specimens were incubated with the primary antibody; detection was carried out with OptiView DAB (ref. 760-700) or UltraView-DAB (ref. 760-500), followed by amplification with Amplification Kit (ref. 760-080) or OptiView Amplification Kit (ref. 760-099). Next the specimens were counterstained with hematoxylin II (ref. 790-2208) and coverslipped. All reagents were obtained from Ventana Medical Systems, Inc. Each slide contained positive, and negative controls. All stainings were perfomed on the VENTANA BenchMark ULTRA.

### Statistical analysis

For the statistical analysis of data from in vitro experiments, the normality assumption hypothesis was tested using Shapiro–Wilk test. Differences between two groups in individual time points were assessed by Student’s t-test or Mann–Whitney *U* test depending on normality of the data in GraphPad Prism® software (LA Jolla, CA). The p-values with *P* < 0.05 were considered to be statistically significant.

## Results

**Fig. 1:**
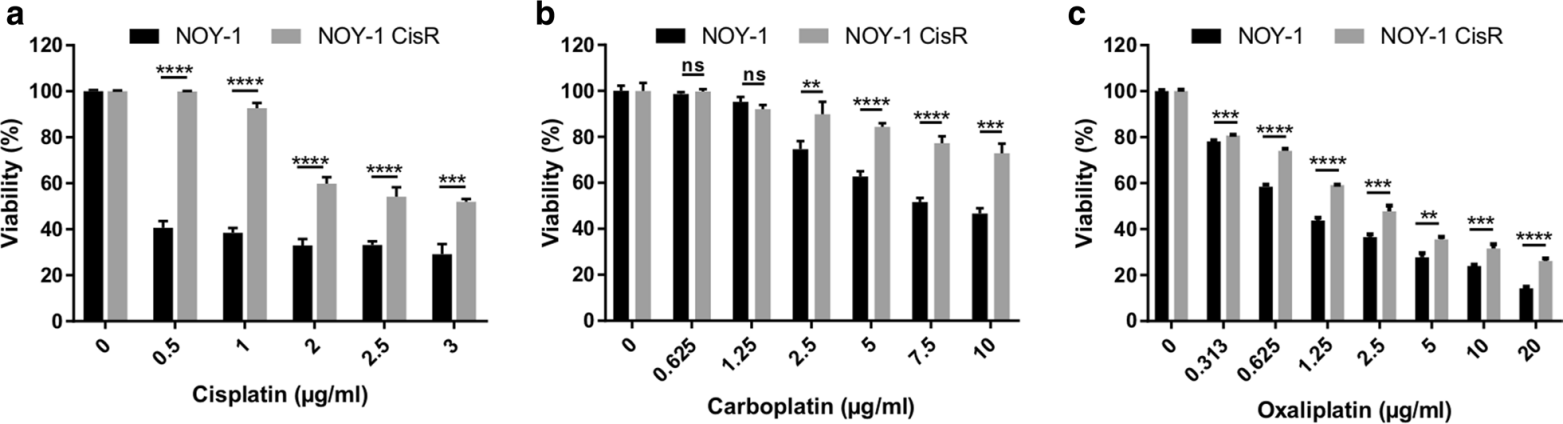
Cisplatin-resistant NOY-1 CisR cell exhibited cross-resistance to platinum drugs. **a**–**c** Cytotoxicity of cisplatin, carboplatin and oxaliplatin in NOY-1 CisR is substantially decreased in comparison to parental cells. Relative viability was determined by luminescent viability assay on day 6 (carboplatin, oxaliplatin) or 7 (cisplatin). Values are expressed as the averages of quadruplicates ± SD. **P < 0.01, ***P < 0.001, ****I < 0.0001

The generated chemoresistant NOY-1 sub cell line, through exposure to gradually increasing concentrations of cisplatin for 6 months, was seven-times more resistant (Fig. 1a; IC_50_ values being 0.34 vs 2.37 μg/ml cisplatin). The resistant phenotype was stable long-term in the absence of cisplatin for at least 3 months (data not shown). The NOY-1 CisR exhibited cross-resistance to platinum analogues, although being less resistant to carboplatin and oxaliplatin (Fig. 1b, c).

**Fig. 2:**
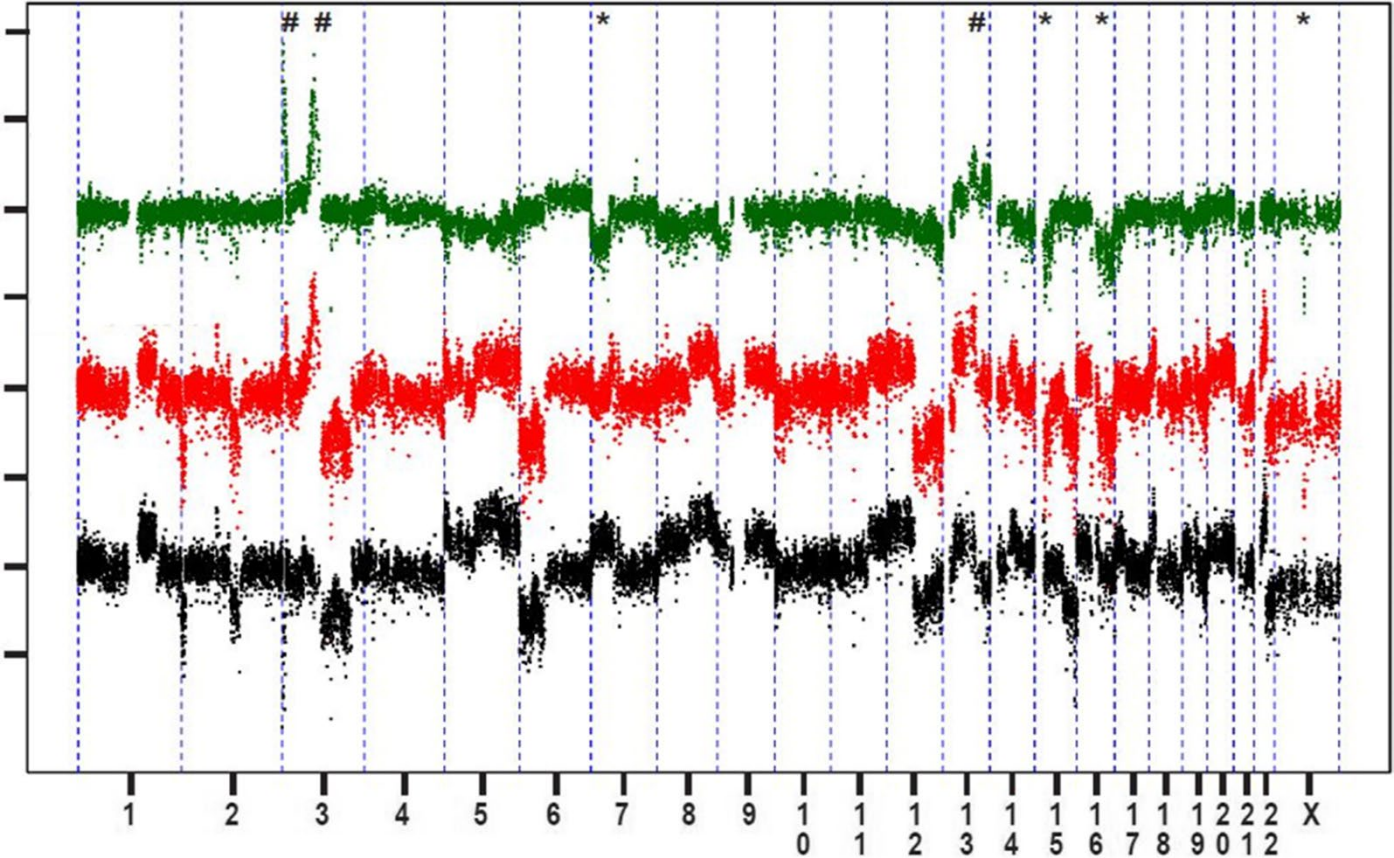
Copy number alterations in the resistant NOY-1 CisR cells. Relative copy number profiles of parental NOY-1 (black color) and cisplatin-resistant NOY-1 CisR cells (red color). Differential copy number profile is shown in green color. Chromosomes are plotted on the x-axis relative to size. ^#^Gain, *Loss

In order to identify potential chemoresistant changes, detailed molecular analysis was performed. Copy number analysis derived from the methylation intensity data (marked in Fig. 2) showed losses on chr7p (3–44 Mb), chr15q (20–40 Mb), chr16q (35–90 Mb), and chrX (70.2–71.6 Mb) and gains on chr3p (tip-9 Mb and 69-86 Mb), and chr13qtel (102–114 Mb). The individual methylation profiles were highly correlated at the CpG site, gene and promoter level (Table 1). The top ranking differentially methylated genes/promotors nearly all exhibited reduced methylation in the resistant cells (Additional file 3. Table S2). A genomic plot of differential methylation at gene and promoter level also shows more hypo-methylation across the genome without an association with copy number changes (Additional file 4. Fig. S1). Differential gene (mRNA) expression was observed for 339 genes > fourfold up-regulated and 639 genes > fourfold down-regulated in the resistant cells. No significant correlation was identified between differential methylation and differential gene expression (Pearson's correlations: 0.007 and − 0.002, respectively, Table 1). The top differentially methylated promoters/genes revealed increased methylation of CAND2 promoter coinciding with four-fold reduced expression in the resistant cells (Additional file 3. Table S2). Reduced gene and promoter methylation in the resistant cells correlated with increased expression of only two genes, ALDH3A1 and RP11-311F12.1 (Clone-based (Vega) gene, GenBank accession number AC005722.1).

**Table 1:** Summary data for the integration of RNAseq and methylation profiling data

Category	Number	NOY-1	NOY-1 CisR
M probes	844,264	0.951	
M prom^&^	34,192	0.965	
M genes^&^	27,021	0.952	
M genes & prom^&^	24,067	0.437	0.417
M diff genes vs M diff prom^&^	24,067	0.653	
R genes (> 0 reads)	29,167	0.949	
R genes (> = 10 reads)^#^	19,157	0.947	
R diff genes (fourfold)^#^	975	Up: 339 Down: 636	
R diff (all/fourfold)^#^ vs M diff genes^&^	16,178/790	0.004/0.095	
R diff (all/fourfold)^#^ vs M diff prom^&^	15,829/758	− 0.044/− 0.013	

*M* methyl CpG beta, *R* RNAseq read counts, *diff* difference between the methyl beta values of wild type NOY-1 and NOY-1 CisR cells or the ratio of the normalized RNAseq read counts. Pearson correlations are provided. ^&^At least 3 probes available within gene or promoter region. ^#^At least 10 RNAseq reads were mapped to individual genes in either or both experiments together

The profile of microRNAs demonstrated that 220/384 microRNAs targets were properly measured, of which 43 targets showed at least four-fold increased or decreased levels after normalization. After quality evaluation, 19 microRNA with differential levels were identified, distributed across the genome (Table 2, Additional file 4. Fig. S1). Overexpression was identified for miR-21, members of the miR-29 family and downregulation for miR-708.

**Table 2:** Cq-values of top differentially expressed microRNAs

microRNA	NOY-1	NOY-1 CisR	Difference^#^
hsa-miR-125a-3p	39.6	32.4	− 7.2
hsa-miR-128-3p	32.1	29.7	− 2.4
hsa-miR-137	31.5	29.2	− 2.3
hsa-miR-143-3p	28.8	32.4	3.5
hsa-miR-199a-3p	34.8	32.3	− 2.5
hsa-miR-205-5p	30.7	39.3	8.7
hsa-miR-21-5p	26.1	23.2	− 2.9
hsa-miR-29a-3p	25.2	22.3	− 2.9
hsa-miR-29b-3p	33.7	29.1	− 4.6
hsa-miR-29c-3p	31.3	29.0	− 2.3
hsa-miR-450a-5p	39.6	31.2	− 8.4
hsa-miR-489	30.1	32.3	2.2
hsa-miR-491-5p	27.3	39.3	12.0
hsa-miR-504-5p	32.9	39.3	6.4
hsa-miR-511-5p	33.5	39.3	5.8
hsa-miR-522-3p	28.9	36.2	7.3
hsa-miR-708-5p	29.6	39.3	9.7
hsa-miR-885-5p	27.3	30.2	2.9
hsa-miR-95-3p	28.1	31.5	3.4

^#^Difference in Cq is given, negative values indicate increased levels in the resistant cells

**Fig. 3:**
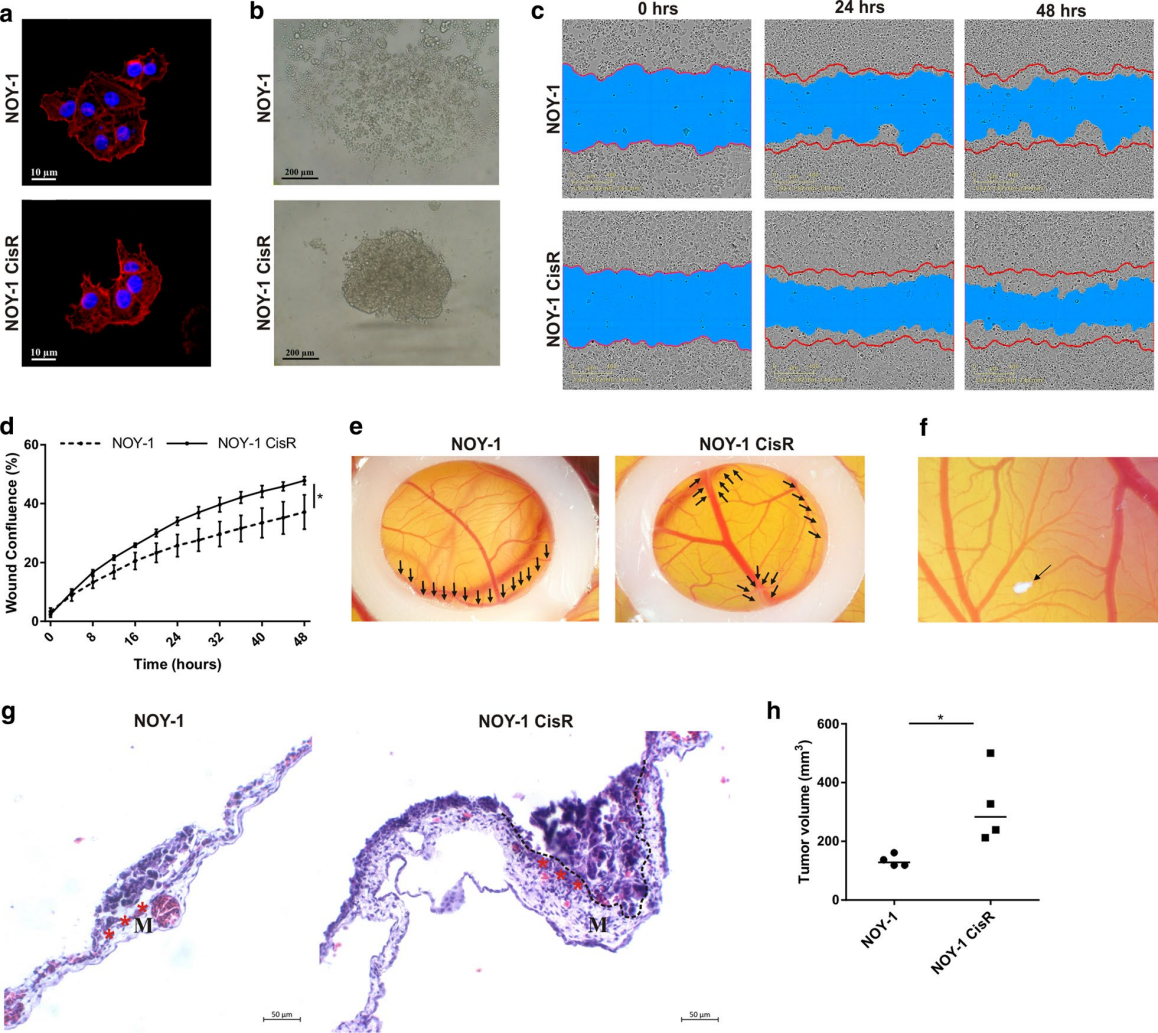
Cisplatin resistance correlated with increased migratory capacity, invasiveness and tumorigenicity, and ability of resistant cells to form 3D multicellular spheroids. **a** Morphology of NOY-1 CisR was similar to parental NOY-1 cells, magnification 630x. **b** NOY-1 CisR cells formed tight 3D multicellular spheroids, when seeded into ultra-low attachment round bottom plates. Parental NOY-1 cells were not able form spheroids in 3D culture conditions, magnification 250x. **c** NOY-1 CisR cells migration was higher than parental NOY-1 cells in a wound healing assay. Confluent monolayers of NOY-1 CisR and NOY-1 cells were wounded and cell migration was observed by live-cell imaging for 48 h. Red line—initial scratch wound line, blue area—wounded area over time. **d** Quantitative evaluation of wound confluence demonstrated significantly increased migratory capacity of cisplatin-resistant NOY-1 CisR cells. Data are expressed as means of three independent measurements each run in quadruplicates ± SD.** e** NOY-1 and NOY-1 CisR cell lines were topically applied into the area defined by a silicone ring and formed micro-tumors in CAM tissue (indicated by arrows). **f** NOY-1 cells produced “micrometastasis” (indicated by arrow) outside the silicone ring. **g** H&E staining of CAMs with tumor cells adhered to ectoderm of the CAMs (marked by asterisks). Resistant NOY-1 CisR cells (outlined by dashed line) invaded into CAM and formed metastatic foci in the mesoderm (M). **h** Subcutaneously injected NOY-1 CisR cells produce bigger xenografts in comparison to NOY-1 cells, data show median tumor volume. 2 × 10^5^ of NOY-1 and NOY-1 CisR cells were injected subcutaneously into the flank of immunodeficient mice, tumor volume was measured regularly. *P < 0.05

No difference in cell morphology (based on α-F-actin immunostaining) was identified between the parental and resistant clone (Fig. 3a). However, the parental NOY-1 cells were not able to propagate in 3D culture conditions, while the resistant variant formed 3D multicellular spheroids (Fig. 3b). In addition, their abilities were investigated in a wound healing assay in vitro, showing a significantly increased migration of the NOY-1 CisR cells after 48 h (Fig. 3c, d). In CAM assay, parental and resistant NOY-1 cells were grafted on quail 7 day embryos cultured *ex ovo* for 3 days. Both cell lines adhered directly to the ectoderm of chorioallantoic membrane and formed micro-tumors (Fig. 3e). Interestingly, NOY-1 CisR cells spread outside of the silicone ring, which was used to limitate the growth of tumor cells on the CAM, and formed small “micrometastasis” (Fig. 3f). NOY-1 CisR cells efficiently penetrated into the mesoderm of CAM in comparison to parental cells which were not able of this invasion (Fig. 3g). Upon subcutaneous injection into nude mice NOY-1 CisR derived tumors reached a median of tumor volume of 283 mm^3^ compared to 128 mm^3^ in parental cells (Fig. 3h), without metastases formation.

**Fig. 4:**
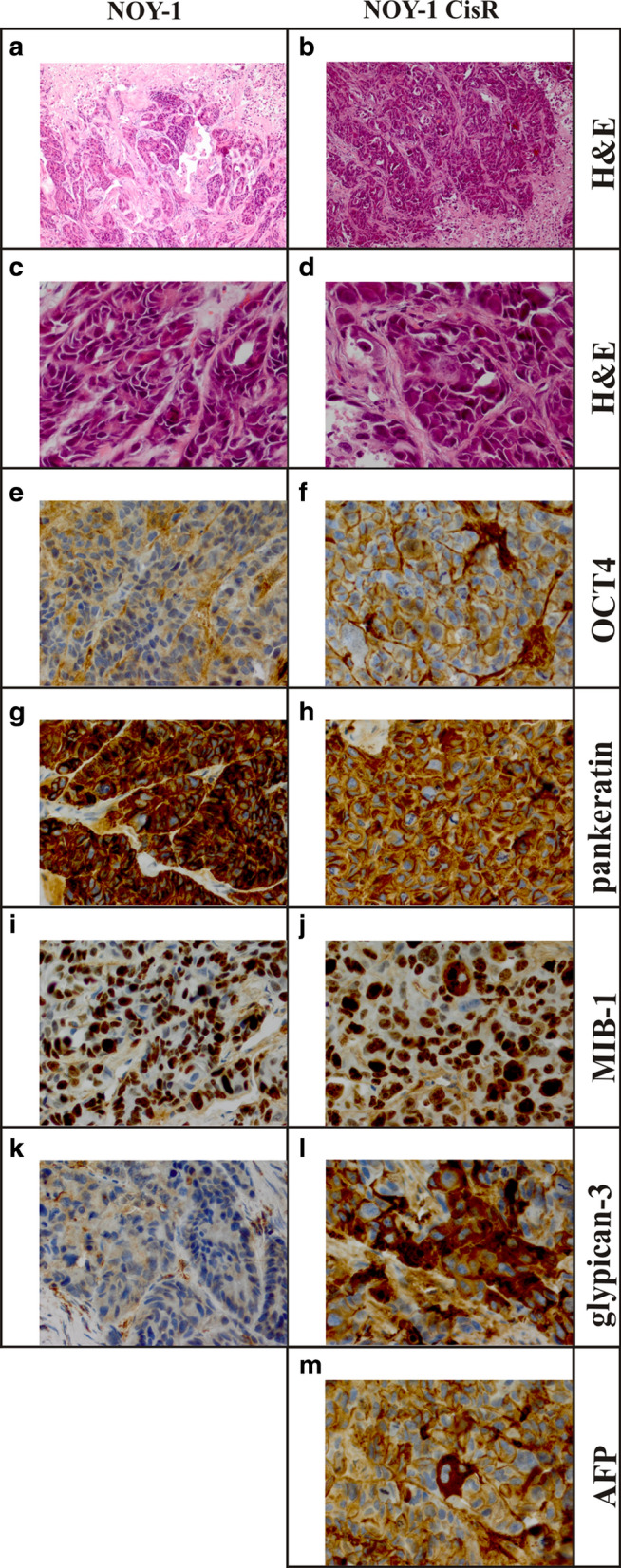
Comparison of morphology and some immunohistochemical characteristics of xenografts derived from parental and cisplatin-resistant NOY-1 cell line. **a**–**d** Hematoxylin and eosin staining showed that both xenografts contain extensive areas of necrosis. The viable tumor tissue focally shows glandular structures in the parental cell line, which are less apparent in the resistant cell line, which has a more solid growth pattern. The biggest difference between the lines is the more severe cyto-nuclear atypia in the resistant cell line, which mainly consists of larger cells, with larger nuclei, and more frequent monstrous nuclei and atypical mitotic figures (magnification: **a**, **b** 100x; **c**, **d** 400x). **e**, **f** OCT4 is negative in the parental and resistant cell lines. The brisk mitotic activity and the large number of atypical mitotic figures are present in resistant cells (magnification 400x). **g**, **h** Both lines strongly express cytokeratins (pankeratin; magnification 400x). **i**, **j** In the MIB-1 staining virtually all nuclei stain positive in the resistant line, whereas in the parental line up to 20% of the nuclei are negative (magnification 400x). **k**, **l** Glypican-3 is not convincingly expressed in the parental line, and focally strongly positive in resistant line (magnification 400x). **m** Only one cell was found unmistakably positive for AFP in the resistant line (magnification 400x), the parental line appears negative for AFP (not shown)

Hematoxylin and eosin staining of xenografts showed that the resistant cells had more cyto-nuclear atypia with a larger range of the size of the cells and nuclei compared to the parental cells, also they had more atypical mitoses (Fig. 4a–d). Both xenografts were negative for OCT4 (Fig. 4e, f) and positive for cytokeratin (Fig. 4g, h). The resistant cell line was strongly positive for MIB-1, whereas in the NOY-1 cells around 80% of the nuclei were positive (Fig. 4i, j). A focally strongly presence of glypican-3 positive cells was found for NOY-1 CisR not convincingly expressed in the parental cells (Fig. 4k, l). Xenograft derived from parental NOY-1 cell line was negative for AFP (data not shown) and only one cell was found positive in the resistant cell line (Fig. 4m).

**Fig. 5:**
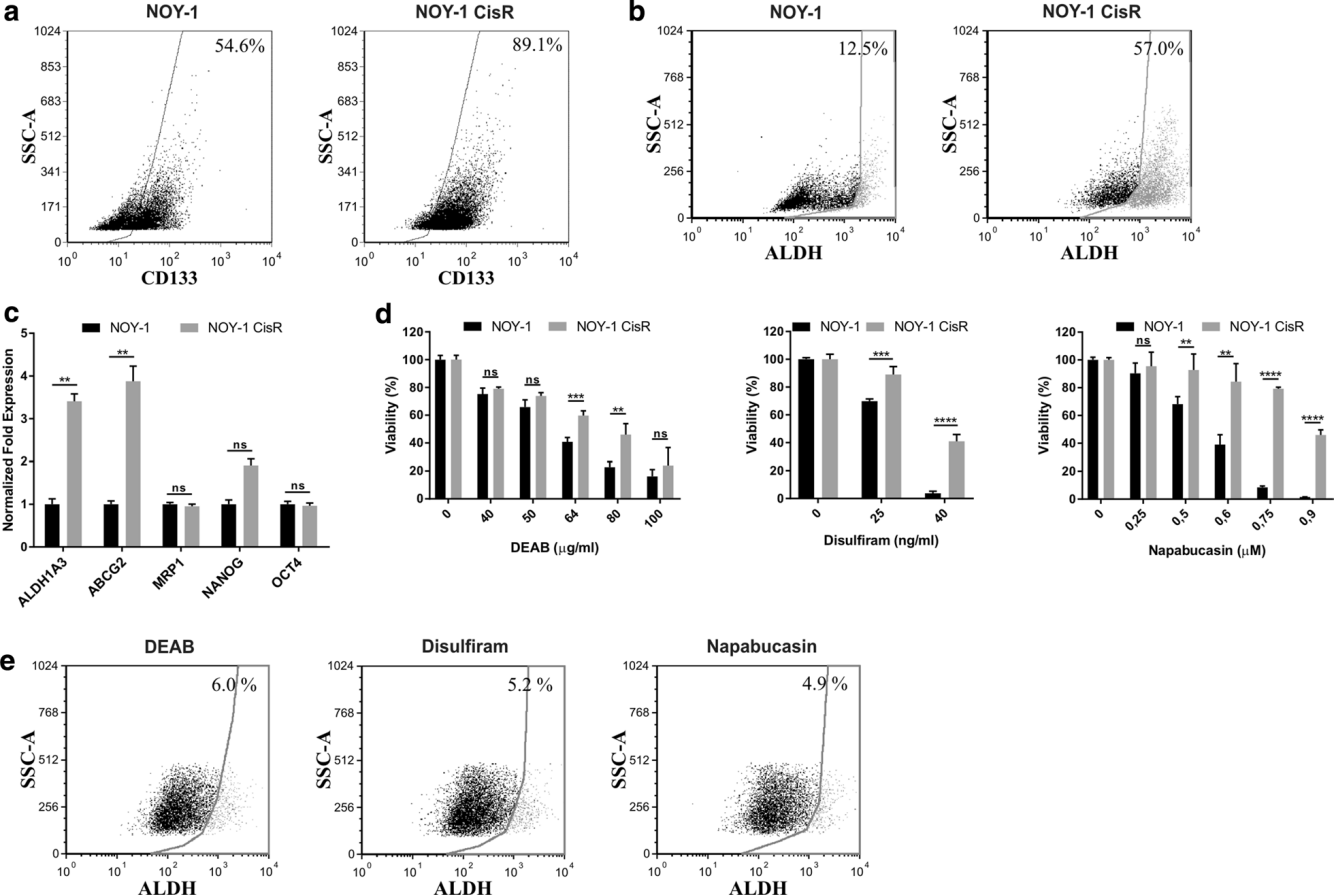
The expression of CSC markers is increased in cisplatin-resistant NOY-1 CisR cells. **a** Increased expression of CD133 marker in NOY-1 CisR cells compared to parental NOY-1 cells was shown in the flow cytometric analysis. Chemoresistant HT-29/EGFP/FUR cells with high CD133 expression were used for the antibody titration and assay setup as a control. **b** The flow cytometry analysis by Aldefluor Assay revealed 4.5-fold increased ALDH activity in NOY-1 CisR cells when compared to parental cells. The number shown in each panel determined the percentage of ALDH + cells. HT-29/EGFP/FUR were used for the assay setup as a positive control. **c** NOY-1 CisR showed higher expression of ALDH1A3 and ABCG2 in expression analysis by qRT-PCR. MSC were used as a positive control for the OCT4 and ABCG2 expression, HT-29/EGFP were used as a positive control for the CD133 and Nanog expression. Chemoresistant HT-29/EGFP/FUR cells were used as a positive control for the ALDH1A3 expression, chemosensitive HT-29/EGFP cells missing ALDH1A3 expression were used as a negative control. **d** NOY-1 CisR cells are significantly resistant to ALDH inhibitors—DEAB and disulfiram, and to inhibitor of STAT3 signaling—napabucasin. Relative viability was determined by luminescent viability assay on day 6. Values are expressed as the averages of quadruplicates ± SD. **e** DEAB (100 µg/ml), disulfiram (200 ng/ml) and napabucasin (4 µM) effectively inhibited overall ALDH activity in NOY-1 CisR cells. The number shown in each panel was determined the percentage of ALDH + cells in Aldefluor Assay. **P < 0.01, ***P < 0.001, ****P < 0.0001

In order to further confirm the association between the cisplatin resistance in chemoresistant oYST cells we performed flow cytometry and expression analysis. In our previous studies [24–27] we have demonstrated high OCT4, MRP1 and ABCG2 expression in MSC, high CD133 and ALDH1A3 levels in chemoresistant HT-29/FUR cells, and NANOG expression in HT-29/EGFP cells. These were used to setup the analysis as suitable controls. Flow cytometric analysis of the CSC marker CD133 showed that NOY-1 CisR showed an enrichment for the CD133 + subpopulation (89.1% of cells relative to 54.6% of parental cells; Fig. 5a). The analysis by functional Aldefluor assay showed a significant difference in total ALDH activity (57.0%-positivity of NOY-1 CisR cells compared to 12.5% in NOY-1; Fig. 5b). Quantitative PCR revealed that the NOY-1 CisR cells exhibited a 3.5-fold increase in the expression of ALDH1A3 and a four-fold increase in the ABCG2 expression, without differences for MRP1, NANOG and OCT4 (Fig. 5c). In line with these findings, NOY-1 CisR cells are also more resistant to inhibitors of ALDH—DEAB and disulfiram, and to a STAT3 inhibitor napabucasin decreasing the ALDH expression compared to the parental NOY-1 cells (Fig. 5d). These inhibitors inhibited overall ALDH activity for more than 50% in NOY-1 CisR cells (57.0 vs 6.0% for DEAB, 5.2% for disulfiram and 4.9% for napabucasin, Fig. 5e).

**Fig. 6:**
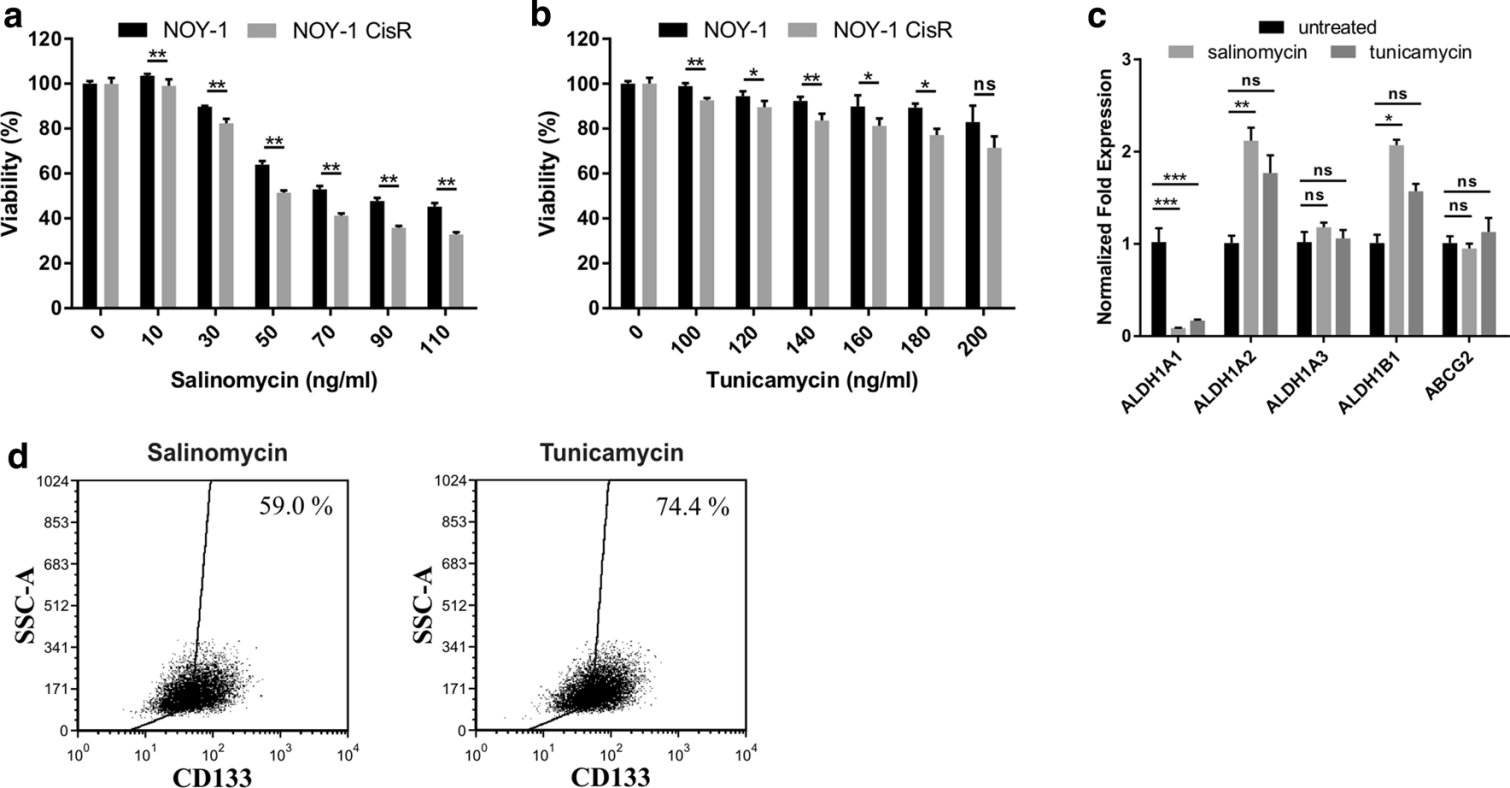
Effect of salinomycin and tunicamycin treatment in cisplatin-resistant NOY-1 CisR cells. **a**, **b** Higher cytotoxic effect of salinomycin and tunicamycin in NOY-1 CisR cells in comparison to parental cells was observed on day 6. **c** Salinomycin and tunicamycin addition downregulated ALDH1A1 in NOY-1 CisR cells, and ALDH1A2 and ALDH1B1 isoforms were overexpressed after salinomycin treatment. MSC were used as a positive control for the ABCG2 expression, HT-29/EGFP cells for the ALDH1A1 expression and chemoresistant HT-29/EGFP/FUR cells were used as a positive control for the ALDH1A2, ALDH1A3 and ALDH1B1 expression. **d** Decreased expression of CD133 was shown in NOY-1 CisR cells treated with salinomycin and tunicamycin in the flow cytometric analysis. *P < 0.05, **P < 0.01, ***P < 0.001

To investigate, whether the resistance to cisplatin can be reversed, the NOY-1 CisR cells were treated with salinomycin (a polyether ionophore antibiotic) or tunicamycin (a glycosylation inhibitor), able to reduce expression of CSC markers and target CSCs in human cancers [35, 36]. Salinomycin significantly inhibited proliferation in the NOY-1 CisR in comparison to parental cells (50% inhibition with 50 ng/ml salinomycin in the NOY-1 CisR and 70 ng/ml salinomycin in parental NOY-1) (Fig. 6a). Tunicamycin addition to NOY-1 CisR cells resulted in a 11% decrease in viability compared to the parental cells (Fig. 6b). Cisplatin did not improve efficiency of these drugs and no synergy was observed (data not shown). Both inhibitors significantly reduced ALDH1A1 expression in contrast to expression of ALDH1A2 and ALDH1B1, which were upregulated after salinomycin treatment. Expression of ALDH1A3 and ABCG2 did not change (Fig. 6c). Flow cytometric analysis of CD133 revelaed also decreased number of CD133 + positive cells after salinomycin (59.0% of positive cells compared to 89.1% in untreated NOY-1 CisR cells) and tunicamycin (74.4% of cells relative to 89.1%) treatment (Fig. 6d).

**Fig. 7:**
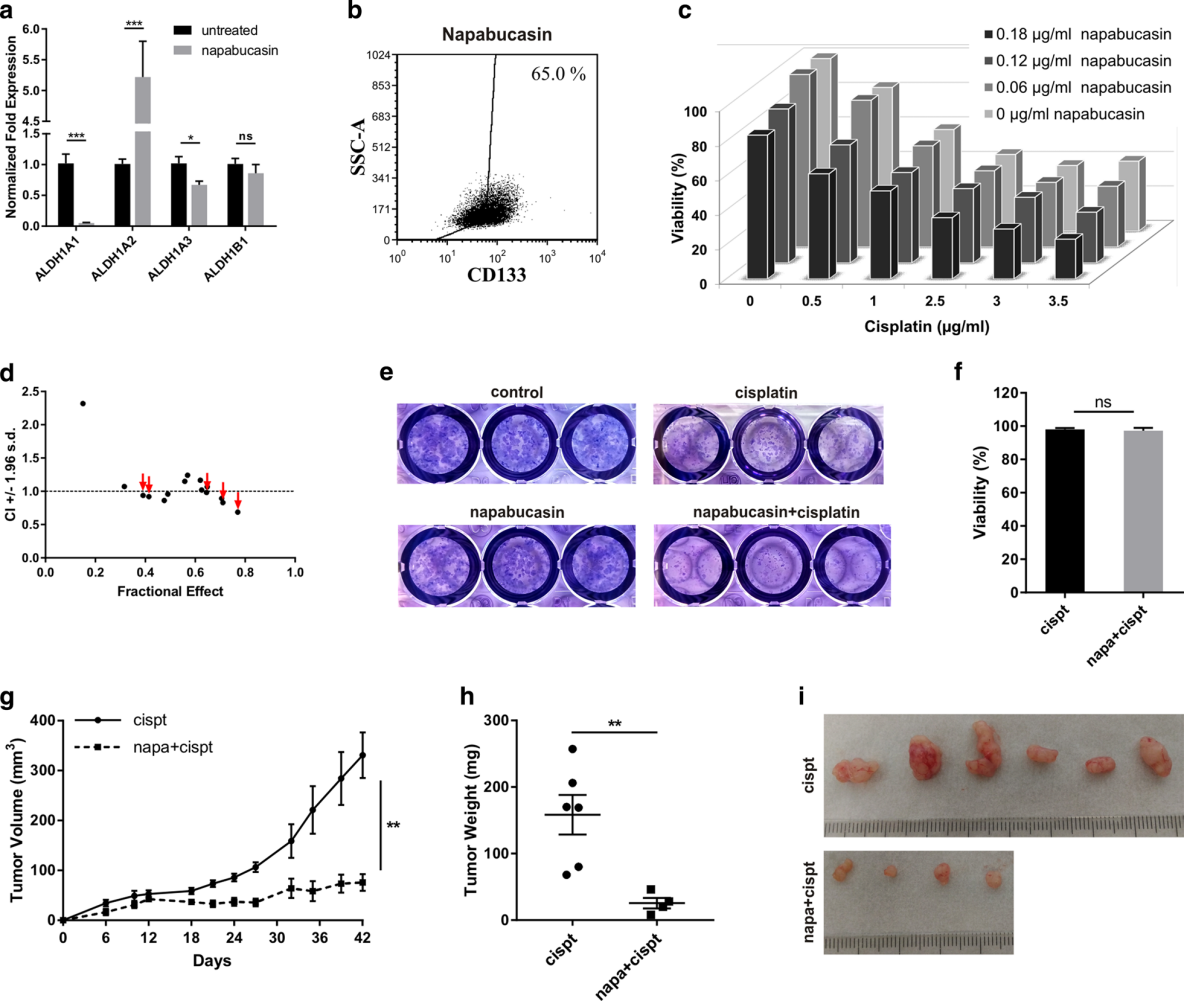
Augmentation of cisplatin treatment by treatment with napabucasin. **a** Napabucasin decreased expression of ALDH1A1 and ALDH1A3 isoforms in NOY-1 CisR cells. ALDH1A2 expression was upregulated after napabucasin treatment. Chemosensitive HT-29/EGFP cells were used as a positive control for the ALDH1A1 expression and chemoresistant HT-29/EGFP/FUR cells as a positive control for the ALDH1A2, ALDH1A3 and ALDH1B1. **b** Flow cytometric analysis revealed decreased expression of CD133 in napabucasin treated NOY-1 CisR cells. **c** Combinatorial treatment with cisplatin and high concentration of napabucasin had synergistic effect. **d** Data obtained by luminescent viability assay were analyzed by Calcusyn software, and Fa-CI plots were created—CI (combination index) on the *y*-axis is a function of effect level (fraction affected, fa) on the *x*-axis (fa = 1−% of viable cells/100). Plots display synergism (CI < 1), additivity (CI = 1) or antagonism (CI > 1) for the entire spectrum of effects [66]. Red arrows indicates the synergy of cisplatin and 0.18 µg/ml napabucasin. **e** Pre-treatment of the cells with napabucasin (0.06 µg/ml) and following treatment with cisplatin (1 µg/ml) reduced the number of clones in a clonogenic assay. **f** Pre-treatment with napabucasin (0.06 µg/ml) did not change viability of cells treated with cisplatin (1 µg/ml). **g** Combination of cisplatin (1 μg/ml) with napabucasin (0.06 µg/ml) significantly inhibited tumor growth in vivo*.* NOY-1 CisR cells were treated with 1 μg/ml cisplatin for six days (group A) or pretreated with 0.06 µg/ml napabucasin and then exposed to 1 μg/ml cisplatin for six days (group B). **h** Tumors were significantly smaller in the group of mice injected with cells pre-treated with napabucasin compared to mice injected with cells treated with cisplatin alone. **i** Image of representative tumors at the end of the experiment showing improved effect of combinatorial treatment. cispt-cisplatin, napa-napabucasin, *P < 0.05, **P < 0.01, ***P < 0.001

In addition, napabucasin, an inhibitor of STAT3 signaling, able to decrease number of ALDH positive cells and sensitize tumor cells to cisplatin, in combination with cisplatin was investigated [37, 38]. Napabucasin significantly decreased expression of ALDH1A1 and ALDH1A3 isoforms. This treatment resulted also in significant upregulation of ALDH1A2 (Fig. 7a). Moreover, subpopulation of CD133 + cells was reduced after napabucasin addition (65.0% of cells relative to 89.1% of untreated NOY-1 CisR cells, Fig. 7b). The combinatorial treatment with cisplatin and 0.18 µg/ml napabucasin showed synergy. However, low concentrations of napabucasin did not increase cisplatin toxicity and an antagnostic effect was observed (Fig. 7c, d). This combination showed no synergy in epithelial ovarian cancer cell lines A2780 and SKOV3, only antagonistic effect was observed (Additional file 5. Fig. S2a–d). Clonogenic assay revealed that a pre-treatment of the cells with 0.06 µg/ml napabucasin and following exposure to 1 μg/ml cisplatin efficiently decreased the number of clones in contrast to the cisplatin or napabucasin treatment alone (Fig. 7e). For the study of napabucasin in vivo, we treated NOY-1 CisR cells similarly as in the clonogenic assay—with combination of napabucasin and cisplatin or with cisplatin alone. Cells in both groups were viable before injection into mice (Fig. 7f). Pre-treatment of cells with napabucasin and following exposure to cisplatin decreased tumorigenicity of NOY-1 CisR cells in vivo compared to cells treated with cisplatin only (Fig. 7g–i).

## Discussion

There is need for suitable models both for the development of novel therapies in the oYST patients and therapeutic strategies for patients with refractory disease. To this end there are only two established human ovarian YST cell lines—NOY-1 and NOY-2 available [39]. The authors generated also a cisplatin-resistant variant (NOY1-CR) by stepwise exposure to cisplatin for 12 months in vitro, resulting in a 22.3-fold higher IC_50_ value after 72 h. A potential for glutathione S-transferase A1 (GSTA1) to become a novel therapeutic target for cisplatin-resistant ovarian YST was also shown [40], of relevance because overexpressed GSTA1 is also reported in cisplatin-resistant ovarian serous papillary carcinomas [41].

The aim of our study was to establish independently a model for evaluation of treatment possibilities in chemoresistant YST and investigate potential therapeutic targets with a focus on the druggable CSC markers. We prepared a cisplatin-resistant subclone (NOY-1 CisR) by cultivation in increasing concentrations of cisplatin for 6 months. The IC_50_ value in our case increased seven-fold, not to be compared directly to the previous study due to the different approaches used to determine chemoresistance. It resulted in significantly higher resistance to the other platinum-based drugs carboplatin and oxaliplatin as well as expected. Cross-resistance to satraplatin and oxaliplatin was described previously and occurred also in cisplatin-resistant TGCTs cell lines resulting in therapeutic failure in overcoming the resistance [42].

An extensive molecular profiling approach was initially used to analyze NOY-1 CisR, followed by a focused analysis on the role of CSC markers, which was not examined in this type of the tumor so far.

To the best of our knowledge, this is the first study describing copy number variations in a chemoresistant YST cell line compared to its parental variant. Alterations in global DNA methylation patterns have been investigated in YST. Even though global hypomethylation of repetitive elements was identified in YST, little hypomethylation of gene regulatory sequences was observed [43, 44]. Hypermethylation at gene promoters in tumors is frequently reported and often linked with transcriptional repression, while loss of methylation may lead to aberrant transcriptional activation [45]. In our model, loss of methylation was correlated with an increased expression of ALDH3A1 isoform in NOY-1 CisR cells. For most genes and gene promoters no correlation was identified, in line with the previous studies.

MicroRNAs (miRNAs) are short non-coding RNAs with a length of ∼22 nucleotides, involved in posttranscriptional regulation of gene expression, playing important role in cancer development and drug resistance [46]. The miR-21, previously described as a potential target to overcome the cisplatin resistance of highly metastatic ovarian cancer tumors, was significantly higher expressed in the NOY-1 CisR subclone [47]. The miR-29 family members (-29a, -29b, -29c) showed increased expression in the resistant cell line (Table 2). It has been shown that miR-29b targets and reduces expression of DNA (cytosine-5)-methyltransferase 3A and 3B in cancer cells, with a subsequent decrease in genome-wide methylation [48]. miR-708 was downregulated in our resistant cells, although a higher expression is reported to be correlated with increased susceptibility of ovarian cancer cells to cisplatin [49]. The differences might be related to various parameters, requiring further investigation. Taken together, molecular analysis of the chemoresistant YST cells unraveled several potential molecular targets, and their role in the cisplatin resistance will be further examined.

Higher migratory capacity, invasion and tumorigenicity were previously demonstrated in various cisplatin-resistant variants derived by similar approaches from the parental cells [50–52]. The morphology with glandular structures, the strong expression of cytokeratins, and the lack of expression of OCT4 are compatible with a YST, as is the expression of glypican-3. The virtually negative AFP does not rule out a YST, while expression of glypican-3 can be found in various somatic tumor types such as hepatocellular carcinoma, hepatoid carcinoma, neuroendocrine carcinoma, thyroid carcinomas, lung carcinoma, liposarcoma, and melanoma [53]. However, the overall described features are also compatible with a somatic type malignancy best classified as a poorly differentiated adenocarcinoma in the parental line. The resistant line seems to have progressed to a less differentiated, more aggressive phenotype, which can be classified as undifferentiated carcinoma. As the parental line has a heterogeneous morphology with patches of similar cells as the cell type which is dominant in the resistant line (see Fig. 4k), it is plausible that the resistant line has developed from this subpopulation in the parental line. Progression to somatic type malignancy is a common mechanism in the development of chemoresistance and it occurs in germ cell tumor types 0, I, II, III, IV and VI [54]. Our data confirmed that NOY-1 cell line is derived from a type II GCT, and that the clinical history of the patient [39] is consistent with an ovarian YST. The lack of expression of AFP and glypican-3 could be due to in vitro changes consistent with the development of a somatic type malignancy, supported by the morphology and IHC on both cell lines.

The subpopulation of chemoresistant cells often overlaps with the cells expressing the CSC markers with a capability of self-renewal and propagation [55, 56]. The CD133 was first identified as a marker of hematopoietic stem cells and since then has been associated with stemness and CSC subpopulation in several cancers, including YST, possibly even being potential target for treatment [57, 58]. Baba et al*.* found that the CD133-positive ovarian cancer cells were more resistant to cisplatin than the CD133-negative cells. These cells had higher tumorigenic potential, they formed tumors that grew to a larger size and appeared with shorter latency than tumors from CD133-negative cells [59]. Our data also confirmed that the development of cisplatin resistance correlates with substantial enrichment in the CD133-positive cell subpopulation in NOY-1 CisR.

High ALDH activity was also previously associated with the CSC phenotype and drug resistance in various chemoresistant models [60–63]. Increased overall ALDH activity in our cisplatin-resistant cells and also higher expression of ALDH3A1 and ALDH1A3 isoforms suggested potential role of this protein family in cisplatin resistance in refractory YST. Despite the fact, that cisplatin-resistant NOY-1 CisR cells were also significantly more resistant to the ALDH inhibitors and napabucasin (STAT3 inhibitor downregulating ALDH expression), these drugs efficiently decreased ALDH activity of resistant cells. Inhibition of the ALDH activity might be a promising therapeutic approach to target the CSCs and to increase the effectiveness of other cancer therapies [26, 27].

Based on the data showing the CSC phenotype in the chemoresistant cells, we decided to examine the potential of the CSC targeting agents to revert the cisplatin resistance in the NOY-1 CisR cells. It has been shown that salinomycin was capable of decreasing the expression of ABCT in multidrug resistant cells, interfering with AKT and Wnt/β-catenin signaling pathway along with increasing sensitivity to cisplatin in the cisplatin-resistant colorectal cells to [64, 65]. Tunicamycin was shown to decrease the expression levels of several CSC markers and reverse the drug resistance in highly resistant hepatocellular carcinomas [35]. Both salinomycin and tunicamycin significantly decreased expression of CSC markers ALDH1A1 and CD133 in NOY-1 CisR cells. More importantly, resistant cells were significantly more sensitive to these inhibitors showing the potential of the CSC targeting strategies to target also refractory YST.

It is of importance for the clinical situation to find the strategies, how to circumvent or revert the cisplatin resistance. One of the approaches may rely on the combinatorial treatment with the drugs potentiating the cisplatin toxicity in synergy or resulting in synthetic lethality in the chemoresistant cells. MacDonagh et al*.* [37] showed that the STAT3 inhibitor napabucasin decreased the ALDH1-positive CSC population of non-small cell lung carcinoma cells whilst decreasing the mRNA expression of critical stemness genes. Reduced expression of CSC markers was observed also in our NOY-1 CisR cells, where napabucasin treatment resulted in downregulation of ALDH1A1, ALDH1A3, CD133 and also decreased overall ALDH activity. Exposure of the cisplatin-resistant non-small cell lung carcinoma cells to the napabucasin in combination with the cisplatin augmented the inhibitory effects observed with the napabucasin alone. Importantly, we observed similar effect in the NOY-1 CisR cells, where combinatorial treatment with the napabucasin and the cisplatin showed synergy and effectively decreased number of the surviving clones in the clonogenic assay. Moreover, NOY-1 CisR cells treated with combination of napabucasin and cisplatin showed significantly lower tumorigenicity in vivo compared to cells treated with cisplatin only. Napabucasin in combination with cisplatin was not successful in the treatment of epithelial ovarian cancer cell lines A2780 and SKOV3 suggesting that this combination could be effective in cell lines with acquired resistance and enriched for CSC markers. These data indicate that there is a possibility to find common combinatorial treatments for different tumor types with the acquired cisplatin resistance resulting in the therapeutic effect.

## Conclusions

In conclusion, we describe here new model for the refractory YST suitable for preclinical drug testing. We have identified several differentially expressed miRNAs and targets associated with the stemness that could lead to better understanding of the mechanisms underlying the cisplatin resistance, unraveling novel biomarkers and discovery of druggable targets. We demonstrated that resistant cells were more sensitive to salinomycin and tunicamycin treatment compared to parental cells. Importantly, combinatorial treatment with the napabucasin augmented the cisplatin toxicity. We suggest here, that these drugs might be used as novel treatment options in order to achieve antitumor effect in the refractory YST patients.

## Supplementary information

**Additional file 1:** Additional methods

**Additional file 2: Table S1** Sequences of primers used for expression analysis.

**Additional file 3: Table S2** Overview of top differentially methylated and expressed genes

**Additional file 4: Figure S1** Chromosomal mapping of differentially methylated gene and promoter DNA regions, and differentially expressed genes and miRNAs of the wild-type NOY-1 and the cisplatin- resistant NOY-1 CisR cells

**Additional file 5: Figure S2** Napabucasin in combination with cisplatin showed antagonistic effect in epithelial ovarian cancer cell lines.

## Data Availability

Not applicable.

## Abbreviations

AFP: Alpha feto-protein
ALDH: Aldehyde dehydrogenase
CisR: Cisplatin-resistant
CSCs: Cancer stem cells
DAPI: 4′, 6-diamidino-2-phenylindole
ECM: Extracellular matrix
FBS: Fetal bovine serum
GSTA1: Glutathione S-transferase A1
IC_50_: Half inhibitory concentration
miRNA: microRNA
MOGCTs: Malignant ovarian germ cell tumors
oYST: Ovarian yolk sac tumor
PBS: Phosphate-buffered saline
RLU: Relative luminiscence units
RT-PCR: Reverse transcription polymerase chain reaction
SD: Standard deviation
TGCTs: Testicular germ cell tumors
