# Supplementary material for: Napabucasin overcomes cisplatin resistance in ovarian germ cell tumor-derived cell line by inhibiting cancer stemness
Source: Cancer Cell Int. 2020 Aug 3;20:364. doi: 10.1186/s12935-020-01458-7 (PMC7397611; doi:10.1186/s12935-020-01458-7)
Supplement: Supplementary file 1 — Additional file 1: Additional methods [file 12935_2020_1458_MOESM1_ESM.pdf]

1.1 Cells

The cisplatin-resistant subclone (NOY-1 CisR) was derived by propagating the cells in increasing concentrations of cisplatin (Hospira UK Ltd, Warwickshire, UK) for six months. Briefly, exponentially growing cells were exposed to 0.05 μg/ml cisplatin initially. When the cells started to expand, the concentrations were gradually increased to 0.3 μg/ml. Expansion was performed until resistant variant exhibited at least four-fold increase in the IC_{50} values for cisplatin and continuously maintained in 0.3 μg/ml cisplatin.

1.2 Methylation profiling

Generation of methylation profiles of cell line genomic DNA isolated using ethanol precipitation was performed at the Erasmus MC Department of Pathology molecular diagnostics lab according to the Illumina protocols (EPIC). Copy number alterations were resolved using the Conumee package (Hovestadt V, Zapatka M. conumee: Enhanced copy-number variation analysis using Illumina DNA methylation arrays. R package version 1.6.0. http://bioconductororg/packages/conumee/. 2015). Differential methylation was identified using the RnBeads package (https://rnbeads.org) using "SWAN" for normalization [1].

1.3 miRNA profiling

Total RNA was prepared using Trizol (Thermo Fisher, USA). miRNAs were converted into cDNA using the specific megaplex primers (ThermoFisher, PN: 4399966) and the reverse transcription kit (ThermoFisher, PN: 3466596) and quantitated on TaqMan Low Density Arrays (384-well Microfluids TLDA card A, ThermoFisher, PN: 4398965) on a TaqMan 7900HT Fast Real-Time PCR Machine using the supplier protocols (ThermoFisher, PN: 4399721). TaqMan miRNA array output data (sds-files) were uploaded in the ThermoFisher Cloud App (https://www.thermofisher.com/nl/en/home/digital-science/thermo-fisher-connect/all-

analysis-modules.html) and analyzed using defined threshold settings for each individual miRNA. Cq values were exported, and globally normalized in Excel.

1.4 RNAseq analysis

Total RNA was prepared using Trizol, DNase-treated (RNeasy Micro Kit, Qiagen, Germany), and quality verified using fragment analysis. The NEBNext Ultra Directional RNA Library Prep Kit for Illumina was used to process the samples. The sample preparation was performed according to the protocol "NEBNext Ultra Directional RNA Library Prep Kit for Illumina" (NEB #E7420). Briefly, rRNA was depleted from total RNA using the rRNA depletion kit (NEB# E6310). After fragmentation of the rRNA reduced RNA, a cDNA synthesis was performed. This was used for ligation with the sequencing adapters and PCR amplification of the resulting product. Clustering and DNA sequencing using the Illumina cBot and HiSeq 4000 was performed according to manufacturer's protocols. The experiments were performed at GenomeScan B.V., Plesmanlaan 1d, 2333 BZ, Leiden. Processing of RNA-seq data was performed using UCSC human genome build hg38 and GENCODE annotation release 28 (GRCh38). FASTQC (v0.11.5) [2] was applied on the paired-end FASTQ files for quality control, both before and after running trimmomatic (v0.36) [3], which removed TrueSeq adapter sequences. STAR (v2.5.3a) [4] was used as aligner, with 2-pass mapping for each sample separately. Mapping quality plot was generated and checked based on sambamba Flagstat (v0.6.7) statistics [5]. Count files, with the number of reads for each gene were created with subread FeatureCounts (v1.5.2) [6].

1.5 α-F-actin immunostaining

Ten thousand of cells growing on microscopic slides for 72 hours were fixed with 4 % paraformaldehyde in PBS for 15 min at room temperature and permeabilized with 0.05 %

Triton-X100 in PBS for 15 min. After overnight incubation with anti-F-actin rhodamine conjugated antibody (1:500; Invitrogen, Life Technology, Slovakia), nuclei were counterstained with DAPI (1:500). Staining patterns were analyzed with a Zeiss fluorescent microscope (AxioImager. Z2, Metafer, Alogo, Ltd., Czech Republic) using Isis upgrade software for Metafer (Alogo, Ltd., Czech Republic).

1.6 Migration assay

Thirty thousand of NOY-1 and NOY-1 CisR cells per well were plated in quadruplicates in ImageLock 96-well plates (Essen BioScience, UK) and let to adhere overnight. Confluent monolayers were wounded with wound making tool (Essen BioScience, UK), washed twice and supplemented with culture medium. Images were taken every two hours for next 48 hours in the IncuCyte ZOOM™ Kinetic Imaging System (Essen BioScience, UK). Cell migration was evaluated by IncuCyte ZOOM™ 2013A software (Essen BioScience, UK) based on the relative wound density measurements and expressed as means of three independent experiments run in quadruplicates ± SD.

1.7 Clonogenic assay

NOY-1 CisR cells were seeded (500 cells per well in 96-well culture plates) and let to adhere in standard culture medium (control) or in medium containing 0.06 μg/ml napabucasin. After 24 hours, cisplatin was added to the cells, and medium-only in control wells. Plates were incubated for 6 days, after which cells were washed and fresh medium was added for another 4 days. Then the cells were stained with May-Grünwald solution.

References

1. Assenov Y, Muller F, Lutsik P, Walter J, Lengauer T, Bock C. Comprehensive analysis of DNA methylation data with RnBeads. Nat Methods. 2014;11(11):1138-40.

2. Schmieder R, Edwards R. Quality control and preprocessing of metagenomic datasets. Bioinformatics. 2011;27(6):863-4.
3. Bolger AM, Lohse M, Usadel B. Trimmomatic: a flexible trimmer for Illumina sequence data. Bioinformatics. 2014;30(15):2114-20.
4. Dobin A, Davis CA, Schlesinger F, Drenkow J, Zaleski C, Jha S, et al. STAR: ultrafast universal RNA-seq aligner. Bioinformatics. 2013;29(1):15-21.
5. Tarasov A, Vilella AJ, Cuppen E, Nijman IJ, Prins P. Sambamba: fast processing of NGS alignment formats. Bioinformatics. 2015;31(12):2032-4.
6. Liao Y, Smyth GK, Shi W. featureCounts: an efficient general purpose program for assigning sequence reads to genomic features. Bioinformatics. 2014;30(7):923-30.
