# Supplementary material for: Napabucasin overcomes cisplatin resistance in ovarian germ cell tumor-derived cell line by inhibiting cancer stemness
Source: Cancer Cell Int. 2020 Aug 3;20:364. doi: 10.1186/s12935-020-01458-7 (PMC7397611; doi:10.1186/s12935-020-01458-7)
Supplement: Supplementary file 2 — Additional file 2: Table S1 Sequences of primers used for expression analysis. [file 12935_2020_1458_MOESM2_ESM.docx]

**Supplementary Table S1.** Sequences of primers used for expression analysis

| **Gene** | **Forward primer (5' to 3')** | **Reverse primer (5' to 3')** | **Product size** |
| --- | --- | --- | --- |
| ALDH1A1 | TTGGAATTTCCCGTTGGTTA | CTGTAGGCCCATAACCAGGA | 182 bp |
| ALDH1A2 | AGGGCAGTTCTTGCAACCATGGAA | CACACACTCCAATGGGTTCATGTC | 193 bp |
| ALDH1A3 | GCCCTTTATCTCGGCTCTCT | CGGTGAAGGCGATCTTGT | 133 bp |
| ALDH1B1 | GCCCCTGTTCAAGTTCAAG | CCTTAAACCCTCCAAATGG | 194 bp |
| OCT4 | ACATCAAAGCTCTGCAGAAAGAACT | CTGAATACCTTCCCAAATAGAACCC | 133 bp |
| NANOG | CAAAGGCAAACAACCCACTT | ATTGTTCCAGGTCTGGTTGC | 346 bp |
| ABCG2 | CGGGTGACTCATCCCAACAT | CAGGATCTCAGGATGCGTGC | 75 bp |
| MRP1 | GCGAGTGTCTCCCTCAAACG | TCCTCACGGTGATGCTGTTC | 118 bp |
| HPRT1 | GGACTAATTATGGACAGGACT | GCTCTTCAGTCTGATAAAATCTAC | 195 bp |
| ACTB | GGACTTCGAGCAAGAGATGG | AGCACTGTGTTGGCGTACAG | 235 bp |
