# Supplementary figures and images for: Napabucasin overcomes cisplatin resistance in ovarian germ cell tumor-derived cell line by inhibiting cancer stemness
Source: Cancer Cell Int. 2020 Aug 3;20:364. doi: 10.1186/s12935-020-01458-7 (PMC7397611; doi:10.1186/s12935-020-01458-7)

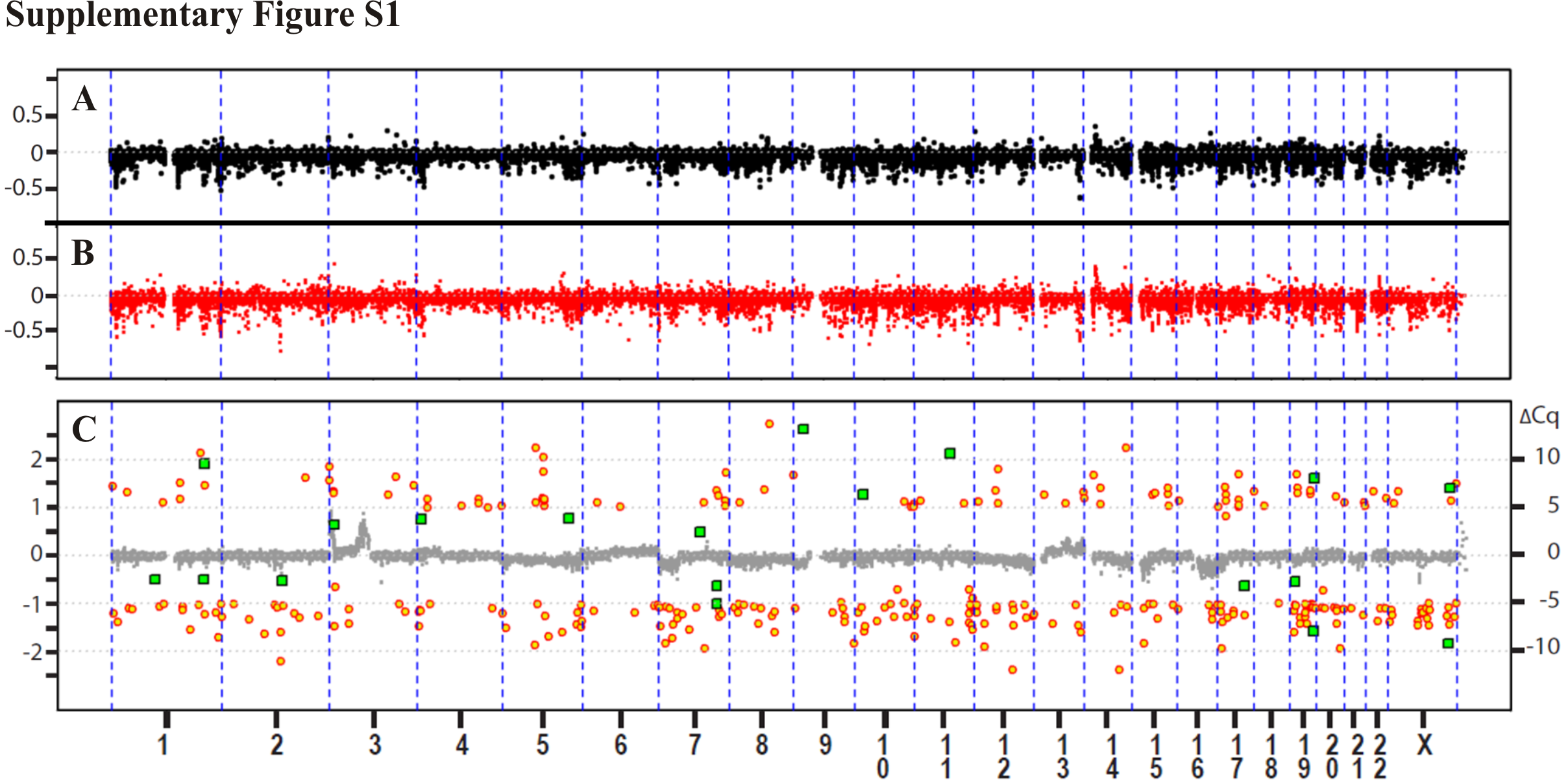

Supplement: Supplementary file 4 — Additional file 4: Figure S1 Chromosomal mapping of differentially methylated gene and promoter DNA regions, and differentially expressed genes and miRNAs of the wild-type NOY-1 and the cisplatin- resistant NOY-1 CisR cells [file 12935_2020_1458_MOESM4_ESM.tif]

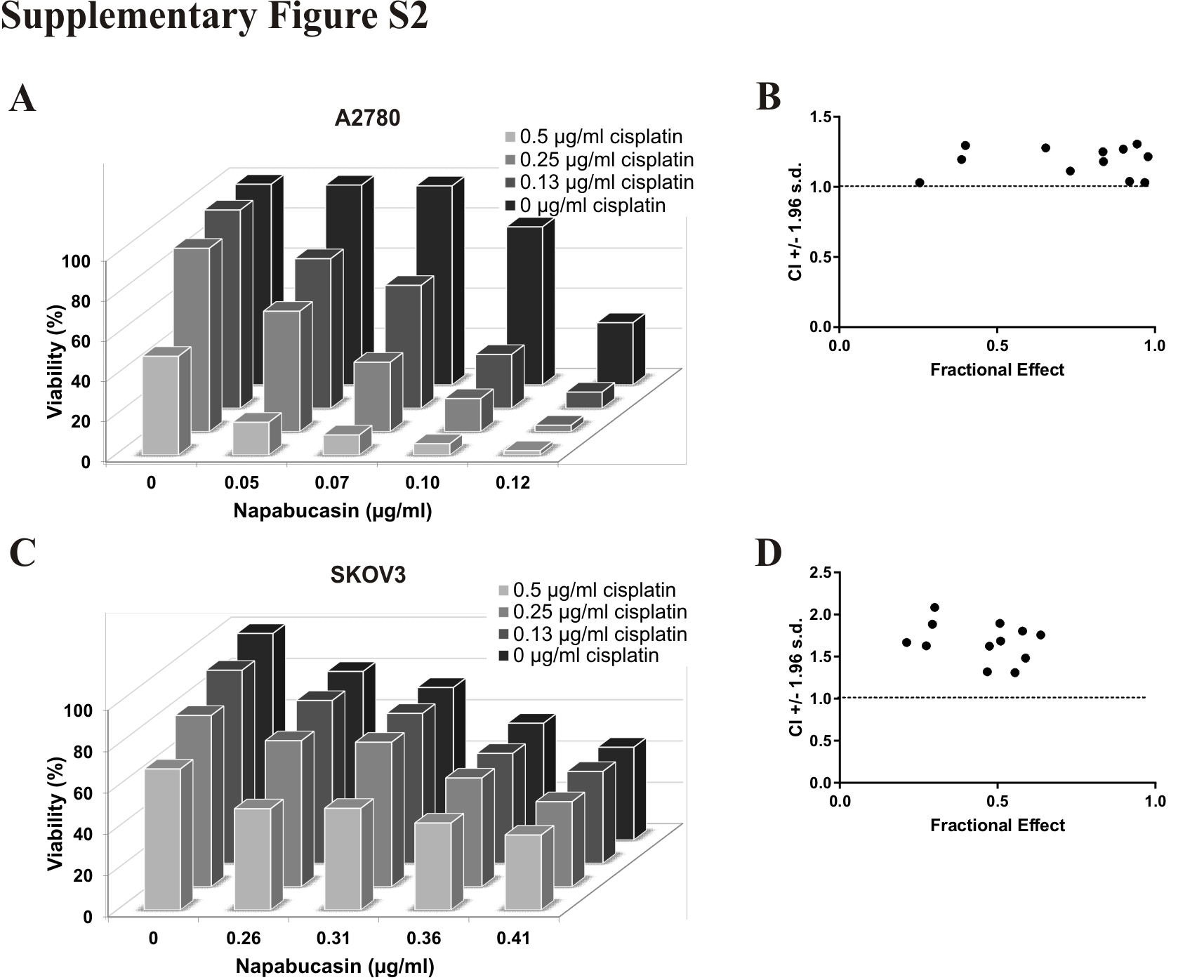

Supplement: Supplementary file 5 — Additional file 5: Figure S2 Napabucasin in combination with cisplatin showed antagonistic effect in epithelial ovarian cancer cell lines. [file 12935_2020_1458_MOESM5_ESM.tif]
